# ATP-Dependent Chromatin Remodeling During Cortical Neurogenesis

**DOI:** 10.3389/fnins.2018.00226

**Published:** 2018-04-09

**Authors:** Godwin Sokpor, Ricardo Castro-Hernandez, Joachim Rosenbusch, Jochen F. Staiger, Tran Tuoc

**Affiliations:** ^1^Institute for Neuroanatomy, University Medical Center, Georg-August-University Goettingen, Goettingen, Germany; ^2^DFG Center for Nanoscale Microscopy and Molecular Physiology of the Brain, Goettingen, Germany

**Keywords:** chromatin remodeling, BAF (mSWI/SNF) complex, ISWI complex, CHD complex, INO80 complex, neurogenesis, neocortex

## Abstract

The generation of individual neurons (neurogenesis) during cortical development occurs in discrete steps that are subtly regulated and orchestrated to ensure normal histogenesis and function of the cortex. Notably, various gene expression programs are known to critically drive many facets of neurogenesis with a high level of specificity during brain development. Typically, precise regulation of gene expression patterns ensures that key events like proliferation and differentiation of neural progenitors, specification of neuronal subtypes, as well as migration and maturation of neurons in the developing cortex occur properly. ATP-dependent chromatin remodeling complexes regulate gene expression through utilization of energy from ATP hydrolysis to reorganize chromatin structure. These chromatin remodeling complexes are characteristically multimeric, with some capable of adopting functionally distinct conformations via subunit reconstitution to perform specific roles in major aspects of cortical neurogenesis. In this review, we highlight the functions of such chromatin remodelers during cortical development. We also bring together various proposed mechanisms by which ATP-dependent chromatin remodelers function individually or in concert, to specifically modulate vital steps in cortical neurogenesis.

## Introduction

Development of the cortex (corticogenesis) is marked by coordination of many key molecular and cellular processes that afford proper brain structure and function. Neurogenesis, one of such cellular events, involves the generation of neurons from neural progenitor cells (NPCs). Embryonic cortical neurogenesis is thus the prenatal aspect of corticogenesis, at which stage the bulk of excitatory (neo)cortical neurons are generated by distinct types of NPCs. Different NPCs can be identified based on their molecular characteristics, morphology, cell lineage commitment, and their site of cell division (Lui et al., 2011; Florio and Huttner, 2014; Taverna et al., 2014; Tuoc et al., 2014; Dehay et al., 2015; Fernández et al., 2016). Apical (APs) and basal progenitors (BPs) constitute the two broad categories of NPCs in the developing cortex. APs include neuroepithelial cells (NEs), apical/ventricular radial glia cells (a/vRGs), apical intermediate progenitors (aIPs) that divide at the apical ventricular zone (VZ) surface. BPs are derived from of APs and include basal/outer radial glia (b/oRG) and basal intermediate progenitors (bIPs). All bRGs lack apical contact, and some lack basal contact. BPs have mitotic figures in the inner/outer subventricular zones (i/oSVZ) (Lui et al., 2011; Dehay et al., 2015).

**Figure 1:**
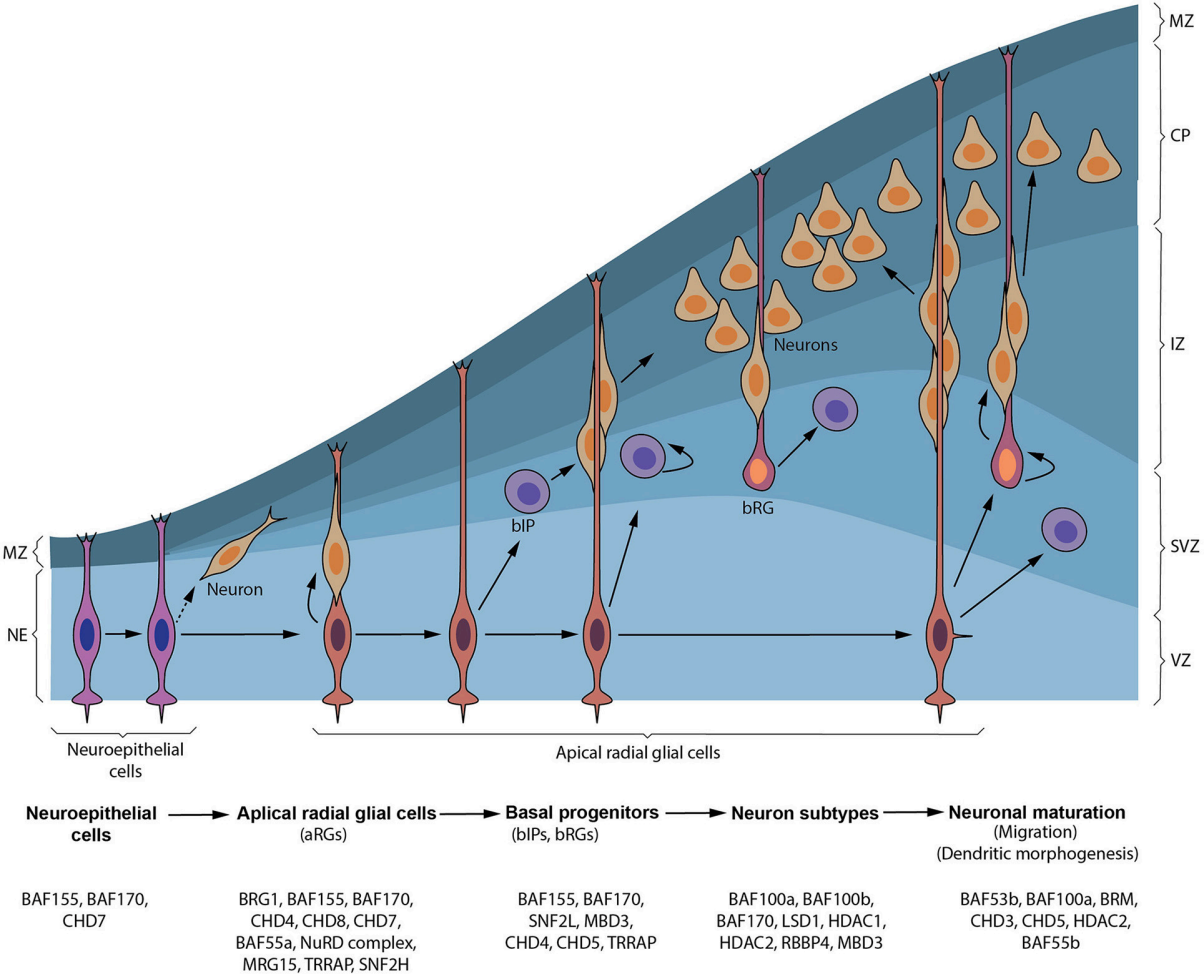
Synopsis of cortical neurogenesis and ATP-dependent chromatin factors involved. Early in cortical neurogenesis, neuroepithelial cells (NEs) divide symmetrically to expand their pool and later divide asymmetrically to give apical NPCs including radial glial cells (RGs) in the ventricular zone (VZ) and pioneer neurons. These apical progenitors proliferate to increase their pool and later divide to give rise to neurons that form the cortical plate (CP) and/or basal progenitors, including basal intermediate precursor (bIP) and basal radial glial (bRG) cells, in the subventricular zone (SVZ). bIP and bRG cells can also self-renew alongside producing neurons before terminally differentiating. Neurons generated from apical progenitors or their progenitor derivatives, migrate predominately to a specified layer in the CP using the long fibers of apical and some basal RG cells as guidance. The marginal zone (MZ) is populated by reelin-producing neurons (Cajal–Retzius [CR] cells) that do not originate from cortical progenitor cells. Chromatin remodeling factors implicated in formation and transition of NEs to apical NPCs and production of basal progenitors through to generation and maturation of neurons during cortical neurogenesis are depicted.

Very early in development of the nervous system, the neural plate and tube are made up of a monolayer of NEs that together form a pseudostratified neuroepithelium and are able to undergo several symmetric divisions to expand their pool. In the part of the neural tube designated to become the telencephalon, commencement of cortical neurogenesis is indicated by the transformation of NEs to aRG and concomitant production of pioneer neurons through asymmetric cell division within a short developmental time window (Figure 1; Götz and Huttner, 2005; Kriegstein and Alvarez-Buylla, 2009; Martínez-Cerdeño and Noctor, 2016). The NE-aRG cell transition is hallmarked by reduction in some epithelial features of NEs such as loss of tight junction complexes and acquisition of astroglial characteristics (Mollgøard and Saunders, 1975; Aaku-Saraste et al., 1996; Hartfuss et al., 2001; Malatesta et al., 2003). By mouse embryonic day 12.5 (E12.5) and gestational week 7 of human development, most NEs are exhaustively converted to aRG cells in the developing cortex (Aaku-Saraste et al., 1996; Hartfuss et al., 2001; Noctor et al., 2002, 2004; Haubensak et al., 2004; Götz and Huttner, 2005; Bystron et al., 2006; Kriegstein and Alvarez-Buylla, 2009; Sahara and O'Leary, 2009).

aRGs are considered as the main cortical NPCs that give rise to the bulk of neurons in the cortical plate (Noctor et al., 2001; Campbell and Götz, 2002; Kriegstein and Götz, 2003; Malatesta et al., 2003; Haubensak et al., 2004; Miyata et al., 2004). This has led to the redefinition aRGs to include their originally perceived limited function as scaffolds for migrating neurons (Levitt and Rakic, 1980; Rakic, 1988; Hatten, 2002). Characteristically, the somas of aRGs reside in the VZ of the developing neocortex, albeit they can undergo what is known as interkinetic nuclear migration therein (reviewed in Taverna and Huttner, 2010). They have short apical and long basal/pial anchorage (Figure 1), display astroglial characteristics, and exhibit increased expression of neuronal genes (Cameron and Rakic, 1991; Bentivoglio and Mazzarello, 1999; Götz and Huttner, 2005; Kriegstein and Alvarez-Buylla, 2009). aRGs can self-renew and/or differentiate directly into neurons (direct neurogenesis) or into BPs which lead to indirect neurogenesis (Figure 1; Götz and Huttner, 2005; Kriegstein and Alvarez-Buylla, 2009; Wilsch-Brauninger et al., 2016). Other structurally and molecularly distinct derivatives of aRGs (i.e., aIPs) collectively called short neural precursors (SNPs) have been found to coexist with aRGs in the proliferative VZ (Gal et al., 2006; Kowalczyk et al., 2009; Stancik et al., 2010). While some aRGs exhaustively convert to neurons after several rounds of cell divisions, others progressively acquire glial progenitor fate and eventually generate cortical glia; thus constituting their developmental switch from neurogenesis to gliogenesis during cortical development (Qian et al., 2000; Costa et al., 2009; Kriegstein and Alvarez-Buylla, 2009; Ge et al., 2012; Magavi et al., 2012).

Normally, after BPs are generated from aRGs via asymmetric division in the VZ, they move to locate in the SVZ (Haubensak et al., 2004; Miyata et al., 2004; Noctor et al., 2004; Kowalczyk et al., 2009). In the developing mouse cortex, a small population of BPs (bIPs) can also self-renew through one or two proliferative division(s), while most of them undergo terminal differentiation to become post-mitotic neurons (Figure 1; Haubensak et al., 2004; Noctor et al., 2004). BPs thus function to increase the neuronal pool in the neocortex since they mediate amplification of neuronal output from individual aRGs. The relative amount of BPs in SVZ varies amongst species, with increasing numbers as the brain gains evolutionary complexity (Martínez-Cerdeño et al., 2006; Arnold et al., 2008; Pontious et al., 2008; Sessa et al., 2008; Nonaka-Kinoshita et al., 2013; Tuoc et al., 2013a).

Particularly, in the ferret and primate neocortices, a highly heterogeneous and dynamic population of basal progenitor (i.e., bRG and bIP) cells are resident in the SVZ or the oSVZ and afford another avenue for increasing neuron output in the developing cortex (Fietz et al., 2010; Hansen et al., 2010; Kelava et al., 2012; Betizeau et al., 2013). Across species, the abundance of basal progenitors, notably bRG, is highly variable and an increased abundance of basal progenitor generation and proliferation during corticogenesis is thought to correlate with increased neuronal numbers, neocortex size and cortical folding during evolution (Lewitus et al., 2013; Borrell and Götz, 2014; de Juan Romero and Borrell, 2017).

When cortical neurons are born in the V/SVZ, they switch on various instructive molecular programs that allow them to mainly migrate by locomotion in a radial orientation using fibers of RG cells for support and guidance (Figure 1). Some early born neurons (Nadarajah et al., 2001; Hawthorne et al., 2010) and bRG cells (Ostrem et al., 2017) have however been reported to radially migrate via somal translocation. A critical step during migration (locomotion) of cortical neurons is the switch in morphology from multipolar to bipolar shape in the intermediate zone (IZ) before finally migrating to their home lamina in the cortical plate (CP) (Noctor et al., 2004; Heng et al., 2008; Evsyukova et al., 2013). Classically, early born cortical neurons migrate to form lower layers whereas late born cortical neurons move to form upper cortical layers. Normally, neurons in the lower cortical layers make extra cortical connections whereas upper layer neurons form connections within the cortex. It is however interesting that the same cohort of primary NPCs generate distinct classes of neurons with upper and lower layer designations. It is becoming increasingly comprehensible that some spatiotemporal factors, including transcriptional and epigenetic factors, play key roles in such subtype specification of cortical neurons (Guillemot, 2007a; Yoo and Crabtree, 2009; Hirabayashi and Gotoh, 2010; Sokpor et al., 2017).

Finally in embryonic cortical neurogenesis, subtype, and areal differentiation processes ensure maturation of neurons so that they can functionally integrate into various cortical circuits in the brain. Usually as part of terminal differentiation and maturation of neurons, there is rapid spouting, pruning and specification of neurites to form either dendrites or a central axon that permit formation of input and output synapses needed for functional development and plasticity of the cortex. As it applies to other discrete steps in cortical neurogenesis, specific molecular factors are known to regulate maturation of neurons during neocortical development (Jan and Jan, 2003; Wu et al., 2007; Chen et al., 2016).

This review essentially gives an overview of important roles of ATP-chromatin remodeling factors during cortical neurogenesis. Detailed information on vital steps of mammalian corticogenesis can be found in other excellent reviews.

Chromatin remodeling complexes are made up of multiple subunits that are assembled in a combinatorial manner to tailor their function to regulating specific developmental events (reviewed in Ho and Crabtree, 2010). They have emerged over the past couple of decades as powerful regulators of many biological processes, including neural development (Yoo and Crabtree, 2009; Hirabayashi and Gotoh, 2010; Narayanan and Tuoc, 2014; Yao et al., 2016; Albert et al., 2017; Sokpor et al., 2017). Accordingly, many genes which encode for chromatin remodelers are found in the developing cortex (Table 1), offering an explanation why their entire ablation or specific subunit inactivation lead to diverse aberrant phenotypes during cortical development (Table 2).

**Table 1:** Expression of genes, encoding for subunits of chromatin remodeling complexes in the developing cortex.

**Subunit**	**Gene**	**E14.5 cortex**		
		**VZ/SVZ**	**IZ**	**CP**
**BAF (SWI/SNF) COMPLEX**				
BAF250a	ARID1A	+++	++	+++
BAF250b	ARID1B	++	+	+++
BAF200	ARID2	+	−	+
BRG1	SMARCA4	+++	+++	++++++
BAF170	SMARCC2	+++++	+++	++
BAF155	SMARCC1	+++	++	+++
BAF180	PBRM1	++	+	−
BAF60a	SMARCD1	+	++	+
BAF60b	SMARCD2	−	−	−
BAF60c	SMARCD3	+++	++	++++
BAF53a	ACTL6A	++	+	−
BAF53b	ACTL6B	−	−	++++
BCL7a	BCL7A	+	+++	+++++
BCL7b	BCL7B	+	−	−
BCL7c	BCL7C	Not found		
BCL11a	BCL11A	+	+++	+++++
BCL11b	BCL11B	−	+	++++
BRD7	BRD7	+++	+++	++
BRD9	BRD9	Not found		
GLTSCR	BICRA	Not found		
GLTSCRL1	BICRAL	Not found		
BAF57	SMARCE1	+	+	+
BAF45a	PHF10	++++	++	++++
BAF45b	DPF1	+	+	++++
BAF45c	DPF3	++	+	++++
BAF45d	DPF2	+++	+	++
SS18	SS18	+	−	−
CREST	SS18L1	++++	+++	++
BAF47	SMARCB1	+++	++	+++++
BRM	SMARCA2	+	−	+++
β-actin	ACTB	++	+	+++
**ISWI COMPLEX**				
CHRAC15	CHRAC1	+	+	−
CHRAC17	POLE3	++	−	+
ACF1	BAZ1A	+++	++	+
SNF2H	SMARCA5	++	+	−
WSTF	BAZ1B	++++	++	++
RSF1	RSF1	+	+	−
TIP5	BAZ2A	+++	+++	+
BPTF	BPTF, FALZ	++	−	−
SNF2L	SMARCA1	−	−	+
RBAP46	RBBP7	+++	+	+
RBAP48	RBBP4	+	−	+
CECR2	*CECR2*	+	−	−
**CHD (NuRD) COMPLEX**				
CHD3	CHD3	+++	+++	+++++
CHD4	CHD4	+++	+	+++
HDAC1	HDAC1	+++	+	++
HDAC2	HDAC2	++	+	+++
MBD2	MBD2	+	−	+
MBD3	MBD3	++	+	++
MTA1	MTA1	++	+	++
MTA2	MTA2	+++	+	++
MTA3	MTA3	+++	+	+++
RBAP46	RBPP7	++	+++	+
RBAP48	RBBP4	+	−	+
**INO80 COMPLEX**				
FLJ20309	INO80D	Not found		
FLJ90652	INO80E	Not found		
MCRS1	MCRS1	+++	−	+
NFRKB	NFRKB	+++++	++++	+++++
UCH37	UCHL5	++	+++	−
AMIDA	TFPT	+	−	+
IES6	INO80C	Not found		
IES2	INO80B	Not found		
ARP5	ACTR5	++	−	−
INO80	INO80	++	−	+
ARP8	ACTR8	+++	+	++
ARP4	ACTL6A	+++	++	+
YY1	YY1	+	−	−
RVB1	RUVBL1	++++	++	+++
RVB2	RUVBL2	+++	++	+++
BRD8	*BRD8*	+	+	++
GAS41	YEATS4	++	+	++
YL1	VPS72	+	−	−
ARP6	ACTR6	−	−	+
ZNHIT1	ZNHIT1	+	−	+++
DMAP1	DMAP1	++	+	++
H2AZ	H2AFZ	+++	+++++	++
SRCAP	*SRCAP*	++	+	++
ING3	ING3	Not found		
EAF6	EAF6	Not found		
MRG15	MORF4L1	+	−	++
β-actin	ACTB	++	+	+++
MRGBP	*MRGBP*	*NA*		
BRD8	*BRD8*	++	+	+++
TIP60	KAT5	++++	++	++++

*VZ, ventricular zone; SVZ, subventricular zone; IZ, intermediate progenitor; CP, cortical plate. –, +, ++, +++, ++++, +++++ indicate relative expression levels (none, weak, moderate, strong, very strong). The expression pattern of most genes was obtained from http://www.genepaint.org (Visel et al., 2004), except ACF1 (Gray et al., 2004), HDAC2 (MacDonald and Roskams, 2008), TIP60 (Thomas et al., 2007)*.

**Table 2:** Function of chromatin remodeling factors during cortical neurogenesis.

**Subunit**	**Mutant**	**Cofactor/target**	**Phenotype**	**References**
**BAF (mSWI/SNF) COMPLEX**				
BAF190a/BRG1	BRG^fl/fl^; Nestin-Cre		Defect in self-renewal and maintenance of murine NPCs	Matsumoto et al., 2006; Lessard et al., 2007; Zhan et al., 2011; Ninkovic et al., 2013
BRM	*BRM^−/−^*	Hdac2, No	Impaired radial migration	Nott et al., 2013
BAF170	*BAF170^*f*/*f*^; Emx1-Cre*	Pax6	Increased genesis of IPs, enhanced cortical volume, surface area and thickness	Tuoc et al., 2013a
	*BAF170^*OE*^*	Pax6	Decreased genesis of IPs, diminished cortical volume, surface area and thickness	Tuoc et al., 2013a
BAF155	*BAF155^−/−^*	Pax6	Abnormal proliferation and differentiation in heterozygotes	Kim et al., 2001
BAF155 BAF170	BAF155^f/f^, BAF170^f/f^ (dcKO); *FoxG1-Cre*	Pax6, Kdm6a/b	Telencephalon is not specified	Narayanan et al., 2015
	BAF155^f/f^, BAF170^f/f^ (dcKO); *Emx1-Cre*		Drastic reduction in cortical thickness	Narayanan et al., 2015
BAF100a	*CTIP1^*fl*/*fl*^; Emx1-Cre; Nex1-Cre*		Specification of subcerebral PNs, reduced Tbr1 and Ctip2 expression, disrupted cortical PN pathfinding	Woodworth et al., 2016
	*Bcl11a^*fl*/*fl*^; Emx1-Cre; Nex-Cre*	Sema3c	Impaired radial migration due to defective multipolar to bipolar morphology, cell accumulation in IZ transition; dysplasia of upper cortical layers	Wiegreffe et al., 2015
BAF100b	*CTIP2^−/−^*		Specification of subcerebral PNs	Arlotta et al., 2005, 2008
BAF55a/ SS18	*SS18^−/−^,SS18^*kd*^*		Defect in closure of neural tube, NPC proliferation, dendritic outgrowth	de Bruijn et al., 2006; Staahl et al., 2013
BAF55b/CREST	*CREST^−/−^*		Defects in dendrite development	Aizawa et al., 2004; Qiu and Ghosh, 2008
BAF53a	*BAF53a^*kd*^*		Impaired neural stem/progenitor proliferation	Lessard et al., 2007
BAF53b	*BAF53b^−/−^*		Defects in dendrite development	Wu et al., 2007
BAF45a	*BAF45a^*kd*^*		Impaired neural stem/progenitor proliferation	Lessard et al., 2007
	*BAF45a^*OE*^*		Extended proliferative phase of cortical neural stem/progenitor cells	Lessard et al., 2007
**ISWI COMPLEX**				
CECR2	*CECR2^−/−^*		Neural tube defects	Banting et al., 2005
SNF2H	*SNF2H^−/−^*		NPC proliferation and differentiation	Alvarez-Saavedra et al., 2014
SNF2L	*SNF2L^−/−^*	FoxG1	Increased cortical progenitor proliferation, more IPs, bigger brain	Yip et al., 2012
**NuRD/CHD COMPLEX**				
CHD3	*CHD3^*kd*^*		Impaired neuronal migration, cell accumulation in lower CP	Nitarska et al., 2016
CHD4	CHD4*^*fl*/*fl*^*; Nestin-Cre		Reduced proliferation of NPCs, increased apoptosis of NPCs, decreased IPs	Nitarska et al., 2016
CHD5	*CHD5^*kd*^*		Impaired neuronal migration, cell accumulation in IZ	Nitarska et al., 2016
		H3K27me3	Accumulation of undifferentiated BPs	Egan et al., 2013
CHD8	*CHD8^*kd*^*	β-catenin	Reduction in NPC self-renewal	Durak et al., 2016
	*CHD8^+/*del*5^*		Increased NPC proliferation	Gompers et al., 2017
HDAC1	*SATB2^−/−^*	Ctip2, Ski1	Specifying the upper layer callosal projection neuron fate over subcerebral projection neuron fate	Alcamo et al., 2008; Britanova et al., 2008
	*SKI^−/−^*	Ctip2, Satb2, Ski1	Specifying upper layer callosal projection neuron fate over subcerebral projection neuron	Baranek et al., 2012
HDAC2	*HDAC2^*kd*^*	Bdnf, No	Neuronal dendritic growth and branching	Nott et al., 2008
	*HDAC2^−/−^*	Protein kinase C, delta	Reduced proliferation of neural progenitors, precocious neuronal differentiation	Hagelkruys et al., 2014
LSD1, HDAC2, RBBP4	LHX2^fl/fl^; Emx1 Cre	Lhx2	Specifying layer 5 Fezf2 and CTIP2-expressing neurons	Muralidharan et al., 2017
MBD3	*MBD3^*fl*/*fl*^; Nestin-Cre*	Smek	Reduced Tbr2+ IPs, reduced cortical thickness, defects in the proper specification of cortical PN subtypes	Knock et al., 2015; Moon et al., 2017
**INO80 COMPLEX**				
TRRAP	*TRRAP^*fl*/*fl*^; Nestin-Cre*	E2f	Reduced apical NPC proliferation, premature production of IPs and neurons	Tapias et al., 2014
MRG15	*MRG15^−/−^*	p21	Decline in neural progenitor cell proliferation and differentiation	Chen et al., 2009, 2011

*NPC, neural progenitor cell; BPs, basal progenitors; IPs, intermediate progenitors; CP, cortical plate; kd, knock-down; del, deletion; dcKO, double conditional knockout, PN, projection neuron*.

**Figure 2:**
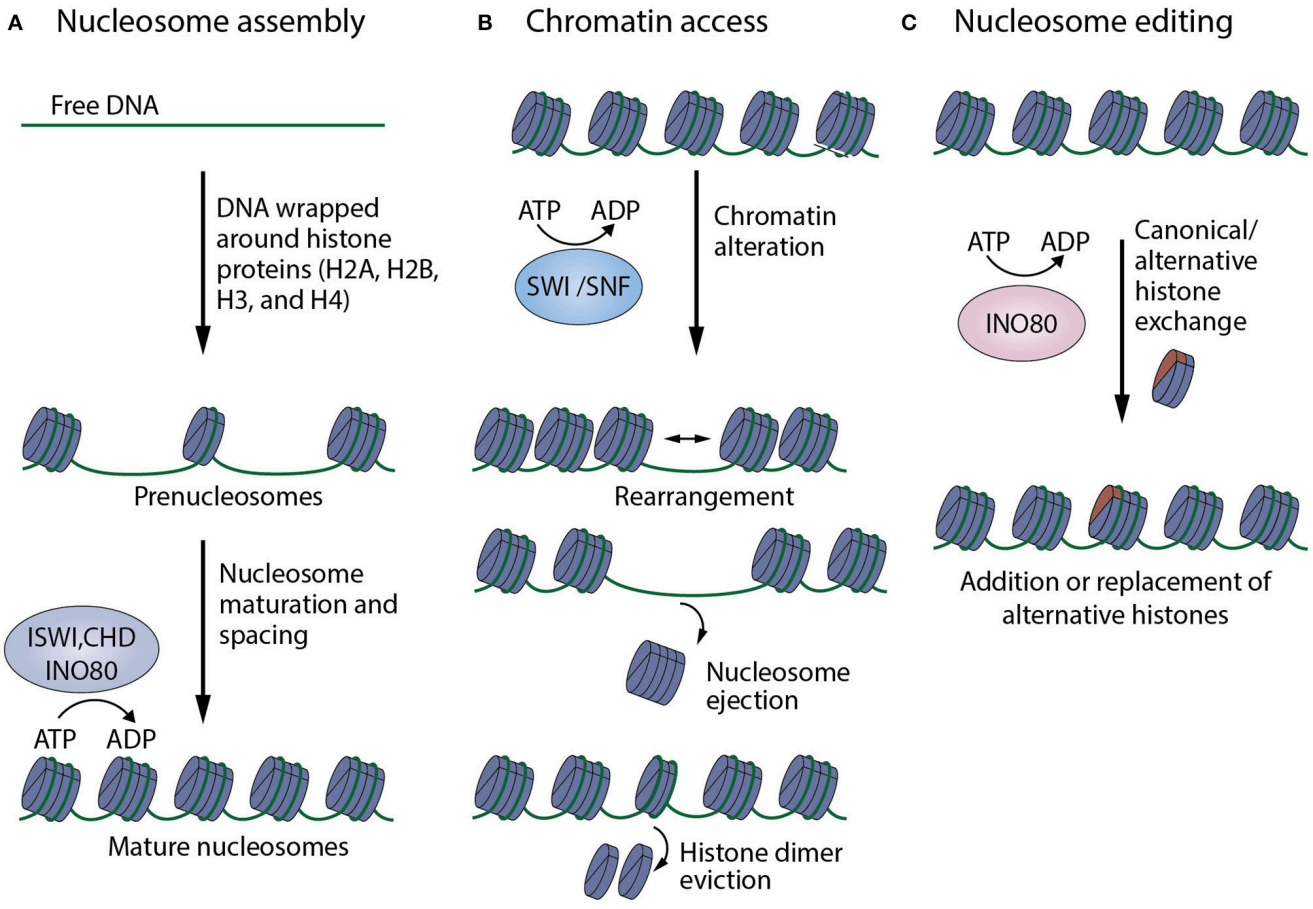
Summary of mechanisms of action of chromatin remodelers. **(A)** Nucleosome organization and assembly: factors, especially those belonging to the ISWI, CHD, INO80 families, are responsible for the random distribution of newly formed nucleosomes, as well as their maturation and arrangement into regularly-spaced chromatin structures. **(B)** Chromatin access: factors that mainly belong to the SWI/SNF family mediate DNA accessibility by nucleosome rearrangement, nucleosome ejection or histone eviction. **(C)** Nucleosome editing: factors of the INO80 family are able to carry out nucleosome editing by promoting the exchange of canonical and variant histones (like H2AZ, shown in red) in chromatin (Adapted from Clapier et al., 2017).

As modulators of chromatin structure, chromatin remodelers exert their effect by influencing gene expression through altering the accessibility of specific DNA regions to transcriptional machinery, and other DNA-binding molecules. Chromatin remodeling subfamilies fall into 3 categories with respect to regulatory strategies they use, namely: nucleosome organization and assembly, chromatin access, and nucleosome editing (Figure 2). Although the modes of chromatin remodeling differ amongst remodeling complexes, there seems to be a common mechanism underlying all chromatin remodeling strategies: DNA translocation (Clapier et al., 2017).

Characteristically, ATP-dependent chromatin remodeling complexes possess ATPase domains that make it possible for them to harness energy from ATP hydrolysis with which chromatin structure reorganization is effected to increase access to DNA. The ATP-dependent mobilization of DNA and its coupling to associated proteins within the remodeling complexes have thus been proposed as the common mechanism across this class of chromatin remodeling factors (Clapier et al., 2017).

**Figure 3:**
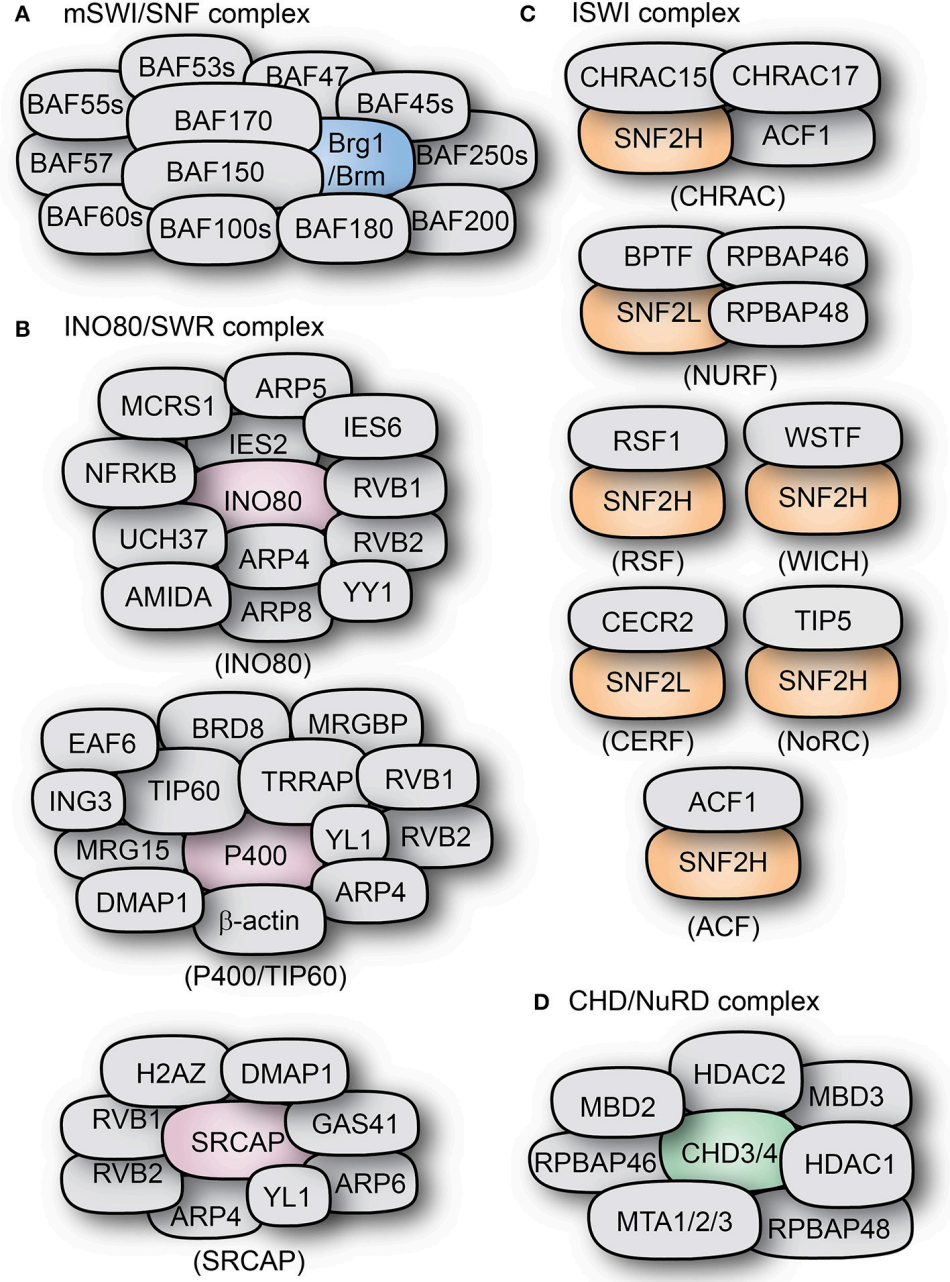
Types and composition of chromatin remodeling complexes. The subunit compositions of some mammalian chromatin remodeling complexes are shown: **(A)** BAF complex, **(B)** INO80/SWR complexes, **(C)** ISWI complexes, and **(D)** the CHD-containing NuRD complex. The core ATPase subunits of the complexes are shown in distinct colors as compared to the other subunits shown in gray color.

Taxonomically, chromatin remodeling factors can be categorized into four subclasses: switch/sucrose non-fermentable (SWI/SNF) complexes, imitation switch (ISWI) complexes, chromodomain helicase DNA-binding (CHD)/Nucleosome Remodeling Deacetylase (NuRD) complexes, and INO80/SWR complexes; based on differences and similarities in their catalytic ATPase domains (Figure 3; Flaus et al., 2006) and associated subunits. The specific modes of action and functional diversity of these specific ATP-dependent chromatin remodeling complexes are discussed further in the next sections.

## Mechanisms of action of chromatin remodeling complexes

### Nucleosome assembly

Remodeling factors within this category are responsible for the maturation of prenucleosomes (early histone-DNA complexes) into octameric mature nucleosomes, as well as the correct spacing of newly formed nucleosomes. This occurs immediately after replication and in association with the replication machinery (Udugama et al., 2011). In general, the assembly of evenly-spaced nucleosomes into heterochromatin silences gene expression (Boyer et al., 2005; Kadoch et al., 2017). Factors responsible for regulating the assembly of nucleosomes belong almost exclusively to the ISWI and CHD subfamilies of proteins. There are some exceptions, like INO80, which has been shown to modulate nucleosome spacing and sliding in an ATP-dependent manner, but not nucleosome disassembly (Figure 2A; Udugama et al., 2011).

Following replication, quickly forming prenucleosomes will provide protection and stability to the freshly synthesized DNA. These prenucleosomes are formed by octameric histone complexes which bind to shorter strands of DNA. These histone-DNA complexes then require the ATP-mediated activity of a motor protein (usually ISWI-related proteins) to produce mature nucleosomes with ~147 bp of DNA associated to them (Becker and Workman, 2013; Fei et al., 2015).

It is believed that multiple factors bind to a region of DNA and promote the translocation of bound DNA along the nucleosomes, in an ATP-dependent manner. These factors move along the DNA strand, pulling neighboring nucleosomes closer to each other until tightly packed, equally distanced arrays are formed (Corona et al., 1999).

### Chromatin access

This strategy is used primarily by members of the BAF (SWI/SNF) subfamily. They make the DNA within the nucleosomes more accessible to other DNA-associated molecules—exposing sites for other proteins (e.g., transcription, repair, or recombination factors) to bind and affect gene expression. They can do so by sliding nucleosomes, evicting nucleosome components (like histone dimers) or completely ejecting full nucleosomes (Figure 2B; Clapier and Cairns, 2014).

Through DNA translocation, this type of remodelers allow the release of longer stretches of linker DNA to be exposed to DNA-binding machinery. By doing so, nucleosomes can translocate near one another, invading the neighboring DNA territory and thus promoting the removal of histone dimers and eventually the expulsion of the complete histone octamer, resulting in nucleosome disassembly (Engeholm et al., 2009) and creating euchromatin state that supports gene expression (Hara and Sancar, 2002; Gong et al., 2006; Ho et al., 2009a; Hu et al., 2011; Tolstorukov et al., 2013). SWI/SNF complexes are known to promote nucleosome eviction in this way, resulting in the removal of a H2A/H2B histone dimer and the further loss of the remaining octamer (Dechassa et al., 2010).

The combinatorial effect of linker DNA translocation and nucleosome disassembly results in a strong increase in DNA availability in the regions targeted by SWI/SNF remodelers. These stretches of newly available DNA are then primed to be targeted by transcriptional machinery, consisting of activators, repressors, or other DNA-binding molecules.

### Nucleosome editing

In particular, nucleosome editing is undertaken by members of the INO80 family of chromatin remodelers (Figure 2C). They mediate the substitution of canonical histones (H2A, H2B, H3, and H4) within an existing nucleosome with alternative histones, in a replication-independent manner. The most prevalent histone variant is H2AZ, which substitutes H2A in H2A/H2B dimers; but other alternative histones exist, like the H3 variants H3.1, H3.2, or H3.3. ATP-dependent remodeling factors mastermind this exchange between canonical and alternative histones. It has been shown that DNA translocation induces mechanical stress within the nucleosome structure, which facilitates the expulsion of classical histone dimers and can promote the incorporation of alternative variants (Clapier et al., 2017).

The presence of alternative variants of H2 or H3 histones is generally associated with nucleosome instability, and nucleosomes containing both of these modifications (H2AZ/H3.3) are enriched in nucleosome-free regions of active promoters in the genome. H2AZ and H3.3 are associated with facilitated access to transcription factors and increased transcriptional activity (Jin et al., 2009).

Although H2AZ is localized all throughout the genome in repressed sites, given its diminished stability, it poises the locus for activation when the associated promoter gets targeted by a transcription factor (Zhang H. et al., 2005). The exact mechanism of alternative histone-dependent transcriptional regulation is not yet clear, but it may involve the regulation of higher chromatin structures (Rege et al., 2015).

## Biochemical and functional diversity of ATP-dependent chromatin remodeling complexes

### SWI/SNF complex

The SWI/SNF superfamily is a class of ATP-dependent chromatin remodelers with homologs in a wide variety of organisms, including mammals (Figure 3A; Ho et al., 2011; Narayanan and Tuoc, 2014; Sokpor et al., 2017). The presence of SWI/SNF remodelers is conserved throughout eukaryotic evolution.

As they are the case in yeast (ySWI/SNF, RSC complexes) and drosophila (BAP, PBAP complexes), the mammalian (m)SWI/SNF complexes are also present in two variants, namely homologous BAF (Brg1/BRM associated factor) and PBAF (Polybromo-associated BAF) complexes. The BAF and PBAF complexes have BRG1/BRM or only BRG1 as their catalytic subunit, respectively (Gangaraju and Bartholomew, 2007). They also differ from each other in the presence of the unique subunits BAF250 in BAF complex, and BAF180 (Polybromo homolog), BAF200 in PBAF complex; reflecting differences in their target specificity (Lemon et al., 2001; Leschziner et al., 2005).

Unlike in drosophila and yeast, mammalian BAF complexes are much more abundant than PBAF (Collins et al., 2002). Also, unlike their fly and yeast counterparts, mammalian BAFs have been found to have a high degree of tissue-specific variability in the subunits that conform the complex (Lessard et al., 2007; Ho et al., 2009a; Bachmann et al., 2016). The different variations of the BAF complex in mammals have been linked to the many biological processes, especially during the development of brain (Matsumoto et al., 2006; Lessard et al., 2007; Wu et al., 2007; Qiu and Ghosh, 2008; Weider et al., 2012; Ninkovic et al., 2013; Tuoc et al., 2013a, 2017; Vogel-Ciernia et al., 2013; Yu et al., 2013; Bischof et al., 2015; Narayanan et al., 2015; Wiegreffe et al., 2015; Bachmann et al., 2016; Nguyen et al., 2016).

SWI/SNF remodelers have been reported to interfere with the structure of chromatin, release of nucleosome-bound DNA, mobilization of DNA along nucleosomes, displacement of histone dimers promoting nucleosome disassembly, among other functions, that facilitate the binding of transcription factors to specific gene loci (Havas et al., 2000; Cairns, 2007; Gangaraju and Bartholomew, 2007). It does so in a step-wise manner, removing first H2A/H2B dimers and then the rest of the histones, releasing the naked DNA (Lorch et al., 2006). SWI/SNF can also remove histone dimers (H2A/H2B) from nucleosomes, independent of its DNA translocation functions (Yang et al., 2007). In addition, recent genome-wide studies indicated that there is enrichment of BAF complexes at promoters (Ho et al., 2009a,b, 2011), and also at super-enhancers (Bossen et al., 2015; Barutcu et al., 2016; Alver et al., 2017; Wang et al., 2017) of active genes involved in cellular processes such as cell proliferation and differentiation.

### ISWI complex

Like many chromatin remodelers, the ATPase ISWI is well conserved across species. ISWI complexes are known to play crucial roles in carrying out nucleosome assembly (Figure 2) as well as nucleosome remodeling and editing (Tsukiyama et al., 1995; Ito et al., 1997). Mechanistically, ISWI binds to the basic patch of the N-terminal tail of histone H4 and also to linker DNA, both of which positively regulate its activity. Binding to linker DNA occurs through the interaction of the C-terminal HAND-SANT-SLIDE (HSS) domain of ISWI. At a catalytic level, the drosophila ISWI is controlled by the action of 2 domains: AutoN and NegC, which negatively regulate ATP hydrolysis and DNA translocation, respectively. ISWI regulation is dependent on the basic patch and linker DNA interaction to AutoN and NegC to remove the catalytic inhibition on the complex, thus promoting DNA translocation and chromatin remodeling (Clapier and Cairns, 2012; Yan et al., 2016).

In mammals, one of the two homologs of ISWI (SNF2H and SNF2L) acts as the catalytic subunit for at least 7 complexes, amongst them are CHRAC, ACF, WICH, RSF, CERF, NoRC, and NURF (Figure 3C; Ito et al., 1997; Varga-Weisz et al., 1997; Strohner et al., 2001; Badenhorst et al., 2002; Cavellán et al., 2006).

#### ACF

In mammals, the basic ACF complex is formed by ACF1 and SNF2H (Figure 3C). ACF can act as a dimer to regulate nucleosome spacing, and does so bidirectionally from the longer to the shorter DNA strand (Racki et al., 2009). The ATPase subunit of ACF, SNF2H, binds as a dimer to both linker DNA and the nucleosome. It uses the HSS domain to sense linker DNA length and through conformational changes coordinates both activities in alternating units of the dimer (Leonard et al., 2015). ACF can also assemble periodic nucleosome arrays *in vitro* in the presence of a histone chaperone (like NAP1 or CAF1). It can also modulate the spacing between nucleosomes, thus changing chromatin accessibility (Ito et al., 1997). Additionally, ACF is also able to further affect chromatin structure by recruiting histone H1 (Lusser et al., 2005).

#### CHRAC

CHRAC is another remodeling complex containing the ATPase ISWI. In mammals, it contains the ISWI homolog SNF2H, as well as ACF1, which are the components of the ACF complex (Varga-Weisz et al., 1997). In addition to them, CHRAC contains two histone-binding proteins: CHRAC15 and CHRAC17 (Figure 3B; Poot et al., 2000). CHRAC is active during chromatin assembly and through its ATP-mediated activity it converts irregular chromatin into chromatin with regularly spaced nucleosomes (Varga-Weisz et al., 1997). Interaction with the tail of histone H4 is essential for CHRAC-mediated nucleosome sliding and ISWI-dependent regular chromosome spacing (Clapier et al., 2001).

#### WICH complex

Functionally similar to ACF, the WICH complex has been associated to the regulation of replication and transcription, as well as the regulation of ribosomal genes. WICH is formed by the interaction of SNF2H and the Williams syndrome transcription factor (WSTF), a protein structurally similar to ACF1 (Figure 3C; Dirscherl and Krebs, 2004). Through WSTF, WICH is able to remodel chromatin at the sites of transcriptionally active rRNA genes promoting transcriptional activation and recruiting histone acetyltransferases like PCAF, p300, and GCN5 (Vintermist et al., 2011).

#### NoRC complex

The complex termed NoRC (nucleolar remodeling complex) is formed by the interaction of the ISWI homolog SNF2H and the protein TIP5 (Figure 3C). It was shown to regulate nucleosome spacing in an ATP- and histone H4-dependent manner (Strohner et al., 2001; Santoro and Grummt, 2005). In addition to its intrinsic role in nucleosome spacing, NoRC can directly and specifically regulate the expression of ribosomal genes due to the interaction of TIP5 with the transcription termination factor TTF-I and the histone deacetylase HDAC1, resulting in transcriptional repression of target genes (Strohner et al., 2001; Zhou et al., 2002; Manelyte et al., 2014).

#### NURF/CERF complex

The NURF complex has the ISWI homolog SNF2L as catalytic subunit, and is involved in transcriptional activation. Human NURF has been found in high levels in the brain, where it can regulate the transcription of genes like Engrailed, suggesting important roles during development (Barak et al., 2003; Li et al., 2016). It accomplishes this by promoting the sliding of histone octamers to release target DNA strands from the nucleosomes, thus increasing DNA availability (Figures 2, 3; Hamiche et al., 1999).

Through the subunit BPTF, NURF can detect trimethylation marks in histone H3 (H3K4me3), targeting these sites for nucleosomal remodeling (Li et al., 2006; Hargreaves and Crabtree, 2011). The N-terminal tail of H4 histone is also essential for the ATPase activity in the NURF complex and the N-terminal tail of H2B is important for the modulation of NURF-dependent nucleosome sliding (Hamiche et al., 2001).

The only other remodeler described so far to incorporate the ISWI homolog SNF2L is the CERF complex. Like NURF, CERF (CECR2 containing factor) is highly expressed in the nervous system and has been associated with regulation of neural development (Lazzaro and Picketts, 2001). The other component of the complex, CECR2 (cat eye syndrome chromosome region candidate 2) is associated to the human disorder cat eye syndrome, and its deletion causes exencephaly in mice (Footz et al., 2001). Together, this complex has nucleosome-dependent ATPase activity and remodels chromatin (Banting et al., 2005).

### CHD/NURD complex

The chromodomain-helicase-DNA binding (CHD) superfamily of proteins is a large class of DNA-binding proteins that can act as chromatin remodeling complexes, and thus regulate gene expression. The members of this class of ATP-dependent chromatin factors are diverse but share a few common characteristics, particularly in the presence of an N-terminal chromodomain, a central SNF2-like helicase motif and a C-terminal DNA-binding domain (Jones et al., 2000). So far, nine members of the CHD superfamily have been described in human (CHD1-9), and these are further classified in three subfamilies (subfamily I-III) according to their structural properties (Hall and Georgel, 2007).

Members of the subfamily I of CHD proteins (CHD1 and CHD2) can act as monomeric units to directly regulate chromatin and transcription (McDaniel et al., 2008; Gaspar-Maia et al., 2009). CHD1, is also directly involved in chromatin assembly and spacing. Monomeric CHD1 associates with histone chaperone NAP1 and catalyzes the addition of nucleosomes to DNA while promoting regular spacing of the resulting nucleosomes. CHD1 remodeling of the chromatin depends on its DNA-binding domains (DBD) to determine the direction of nucleosome sliding and the length of internucleosomal DNA (McKnight et al., 2011).

The subfamily II of CHD proteins (CHD3, CHD4) are core catalytic components (ATPases) of the nucleosome remodeling deacetylase NuRD complex (Figure 3C). They can directly bind to the histone deacetylases HDAC1 and HDAC2, as well as the DNA-binding proteins MDB2 and MDB3 and MTA1, MTA2, and MTA3 (Schmidt and Schreiber, 1999; Marhold et al., 2004; Le Guezennec et al., 2006). Like many chromatin remodeling complexes, NuRD can have a highly variable subunit composition which confers functional specificity to the complex in a tissue-dependent manner (Feng et al., 2002; Lai and Wade, 2011). NuRD activity has been mainly associated to transcriptional repression (Hirose et al., 2002; Srinivasan et al., 2006). It can bind directly to methylated DNA and to transcription factors (like the Polycomb group) and promote histone deacetylation in addition to its default chromatin remodeling activity; hence making NuRD a powerful regulator of chromatin structure and gene expression (Hendrich and Bird, 1998; Kehle et al., 1998; Wolffe et al., 1999; Zhang L. et al., 2005).

Subfamily III (CHD5-9) contains recently discovered members that have few known interaction partners. But like the other CHD proteins, they seem to be associated with chromatin structure and remodeling (Hall and Georgel, 2007).

### INO80/SWR complex

Although diverse, the members of the INO80 subfamily of chromatin remodelers are characterized by a central split ATPase domain subunit and the presence of two RuvB-like helicases, Rvb1 and Rvb2 (Figure 3B). Despite their core functional similarities, the incorporation of additional subunits to the INO80 complexes (most importantly INO80, SRCAP, and P400/TIP60) can confer very different roles in chromatin remodeling, nucleosome modification, and gene regulation.

#### INO80

By itself, the INO80 complex acts as a nucleosome spacing factor, promoting the generation of regularly spaced nucleosomal arrays (Yen et al., 2012; Gerhold and Gasser, 2014). This INO80-mediated chromatin remodeling modulates gene transcription, both as an activator and as a repressor (Morrison and Shen, 2009; Hogan et al., 2010). INO80 can also control the levels of H2AZ in transcriptionally active sites, by catalyzing the exchange of H2AZ/H2B dimers in the chromatin with free H2A/H2B. The interaction and recognition between INO80 and deacetylated H2AZ is essential for the maintenance of genome integrity (Papamichos-Chronakis et al., 2011). INO80's ATPase activity, DNA binding, and mobilization are dependent on regulation by associated ARPs, namely Arp8 and Arp5, which can also act as histone chaperones (Shen et al., 2003).

#### SWR1/SRCAP

In yeast, Swr1 is the catalytic subunit of the complex SWR-C that exchanges conventional H2A histones with H2AZ in specific locations in the genome, thus regulating gene expression. The exchange of histone varieties occurs between a H2A/H2B dimer and a H2AZ/H2B dimer with Swr1 acting as a histone chaperone (Mizuguchi et al., 2004; Hong et al., 2014). Swr1's closest ortholog in the human is the Snf-2 related CREB-binding protein activator protein (SRCAP) (Figure 3B) which acts as a coactivator for many transcription factors known to interact with CBP. As Swr1 does, SRCAP acts like the ATP-dependent catalytic subunit of its own complex which substitutes H2A-containing histone dimers with H2AZ variants, thus acting as coactivator (Ruhl et al., 2006).

H2AZ can be found all throughout the genome, flanking nucleosome-free regions. It is present at both, active and repressed genes. Addition of H2AZ is promoted by acetylation of the tails of histones H3 and H4 and the protein Bdf1, a component of the SWR1 complex (Raisner et al., 2005). Swr1's activity is positively regulated by the presence of H2A-containing nucleosomes, as well as by the presence of free H2AZ (Luk et al., 2010). Histone H3 has also variants (namely H3.1, H3.2, and H3.3) which differ slightly in amino acid sequence and regulation (Hake et al., 2006). H3.3 for instance is present in transcriptionally active genes and can be incorporated into nucleosomes in a replication-dependent or independent manner. Interestingly, acetylation of histone H3 promotes H2AZ or H2A exchange from the nucleosomes (Watanabe et al., 2013).

#### TIP60–P400 complex

TIP60 has been described as an acetyltransferase capable of acetylating core histones H2A, H3, and H4 as well as transcription factors and signaling molecules, regulating gene expression and modulating cellular responses (Halkidou et al., 2004; Sun et al., 2005; Sapountzi et al., 2006). In most of its biological roles, TIP60 can be found in association with various interaction partners forming transient complexes, but in cases of transcriptional regulation and DNA repair, it exists as a part of a stable multicomponent complex with at least 18 subunits (Sapountzi et al., 2006). Key components of this complex include the scaffolding protein TTRAP and the chromatin remodeling ATPase P400 (or Domino), as well as shared components with the SRCAP complex, like Rvb1 and 2, or Arps (Figure 3B; Ikura et al., 2000). The activity of the TIP60/P400 complex has been associated with many developmental processes (Ueda et al., 2007; Wu et al., 2007; Fazzio et al., 2008).

As a chromatin remodeler, TIP60/P400 complex acts mainly in response to DNA damage by detecting the affected sites and promoting the remodeling of neighboring chromatin into an “open” state, through the acetylation of histone H4 and the selective exchange of histone H2A variants (Ikura et al., 2000; Kusch et al., 2004; Tamburini and Tyler, 2005). This allows the repair machinery to efficiently access sites of double strand breaks in DNA and exert its function.

## Function of ATP-dependent chromatin remodeling complexes during cortical neurogenesis

Although most multimeric ATP-dependent chromatin remodeling factors are ubiquitously expressed, many specific functional variants of such complexes can be formed depending on the tissue- and/or cell-type involved. The functional plurality and specificity of such chromatin remodeling complexes can also be triggered by specific developmental cellular demands (e.g., DNA repair, proliferation, cell death, differentiation, maturation). It has been commonly proposed that some chromatin remodel complexes, such as the BAF and NuRD complexes, can be functionally specified by reconstituting or reshuffling some of their subunits to configure the entire complex toward specific ontogenetic functions. The existence of polymorphic or paralogous forms of the subunits of these ATP-dependent chromatin remodeling factors thus allow for some plasticity of their related complexes to customize their overall functional activity.

For example, as recently reviewed in Sokpor et al. (2017), the BAF complex, which is known to be composed of at least 15, varies in composition as pluripotent embryonic stem cells (ESCs) acquire multipotency to become NPCs that subsequently differentiate into neurons during neural tissue development. The embryonic stem cell BAF (esBAF) complex contains the following BAF complex subuints: BAF60a/b, BAF155, BAF250a, and BRG1 but not their polymorphic or paralogous forms: BAF60c, BAF170, and BAF250b and BRM, respectively.

The neural progenitor BAF (npBAF) complex formed in NPCs as ESCs acquire multipotent NPC fate, is distinctively composed of high amounts of BAF155, low levels of BAF170, BAF250a/b, and BRG1 or BRM ATPase. However, subunits like BAF45a/d, BAF53a, and BAF55a in the esBAF complex are maintained in the npBAF complex. On the other hand, the reconstitution of the npBAF complex to form the neuronal BAF (nBAF) complex during differentiation of NPCs into neurons entails substituting BAF45a, BAF53a, and BAF55a for BAF45b/c, BAF53b, and BAF55b, respectively, and alongside low levels of BAF155 and high amounts of BAF170. The combinatorial assembly of the BAF complex is also elegantly reviewed in Ho and Crabtree (2010) and that of the NuRD complex is described in Feng et al. (2002); Denslow and Wade (2007); Lai and Wade (2011).

As previously mentioned, neocortical development comprises specific developmental processes such as neural specification and patterning, establishment and subsequent transformation of NEs to RGs, proliferation and differentiation of neural progenitors, neuronal subtype specification and migration, neuronal maturation, and ultimate integration of neurons into maturating functional cortical circuits. By applying *in vivo* animal models and neural progenitor culture systems *in vitro*, these specific events have been investigated to elucidate various epigenetic mechanisms regulating them. In the following subsections, we put together various studies that implicate specific ATP-dependent chromatin remodeling factors in controlling the aforementioned aspects of cortical neurogenesis.

### Neural specification and patterning

The induction and areal specification of neural tissue are fundamental processes responsible for the development of the nervous system. Through the orchestration of several morphogenetic elements, including signaling and transcriptional factors, specific aspects of the simple embryonic ectoderm receive instructions to progressively transform into more complex neural structures in the course of development (Muñoz-Sanjuán and Brivanlou, 2002; Schohl and Fagotto, 2002; De Robertis and Kuroda, 2004; Wilson and Houart, 2004; O'Leary et al., 2007).

The chromatin remodeling BAF complex appears to be an integral part of the regulatory cascade that determines specification and formation of the cortex and the entire nervous system (reviewed in Sokpor et al., 2017). This assertion is backed by experiments in which the entire BAF complex was conditionally ablated by knockout of the scaffolding subunits BAF155 and BAF170 under the control of the early acting (E8.5-9.0) Foxg1-Cre driver line in the emerging telencephalon (Narayanan et al., 2015; Bachmann et al., 2016; Nguyen et al., 2016). Notably, the deletion of BAF complex from early telencephalic domains absolutely abolished specification of the cortex as well as other head structures (Narayanan et al., 2015; Bachmann et al., 2016; Nguyen et al., 2016). The forebrain was however specified when the BAF complex functionality was lost conditionally at a later embryonic stage (~E11.5) under the control of the Emx1-Cre promoter. However, such mutant mouse brains presented with extreme abnormalities that likely could not support normal cortical functions (Narayanan et al., 2015; Nguyen et al., 2016).

SRG3 (SWI3-related gene product), a mouse homolog of the human BAF complex subunit BAF155, has been shown to be essential in the specification and spatial patterning of telencephalic regions of the developing brain. About 20% of mice heterozygous for SRG3 displayed abnormal location and formation of the forebrain, a condition called exencephaly (Kim et al., 2001), which is generally caused by failure in the elevation of the neural fold and subsequent expansion of neural tissue. Specifically, by performing *in situ* hybridization in E12.5 exencephalic SRG3 heterozygous embryos with BF-1 (brain factor-1)/Foxg1 probe, it was observed that the forebrain neuroepithelium was abnormally located underneath the thalamus. Examination of the SRG3 mutant head structures at E16.5 revealed several anomalies, including gross morphological malformation of the cerebral cortex and other forebrain structures. An elevated expression level of SRG3 protein early in the development of the telencephalon, that is during neural tube closure (E8.5–E9.5), and the sustenance of its constitutive high expression level in the central nervous system seem to be critical for the proper specification and development of the cortex (Kim et al., 2001).

To further support the role BAF complex in cortical specification and patterning, it has been shown in one study that the transcription factor Ctip1 (BAF100a/Bcl11a), which is also a variant subunit of the BAF complex, is a powerful morphogen in sensorimotor area specification and patterning during neocortical neurogenesis (Greig et al., 2016). Ctip1 was found to be an indispensable factor that operates in newly generated cortical neurons to control acquisition of sensory identity. This is mainly achieved through its role in establishing sensory-specific gene expression patterns for output circuitry, and also formation of sensory maps for thalamocortical inputs (Greig et al., 2016). Loss of Ctip1 function severely disrupted the molecular differentiation in primary cortical sensory areas likely due to downregulation of relevant gene expression programs and ectopic expression of motor cortex-specifying genes. This implies that, Ctip1 suppresses motor identity of projection neurons in primary sensory areas of the cortex, thereby contributing to creation of the molecular boundaries that parcel various functional cortical areas (Greig et al., 2016). The precise role of Ctip1 in the specification and connection of projection neurons will be subsequently discussed under the subheading “generation of neuronal subtype.”

Put together, our knowledge of the involvement of epigenetic morphogens like ATP-dependent chromatin remodeling factors in determining the ultimate relative volume and location of functional domains of the neocortex during corticogenesis is expanding. More mechanistic details should be unraveled in further investigations to deepen our current understanding.

### Expansion and maintenance of apical neural progenitor pool

As previously discussed, the developmental transformation of NEs to RGs is a critical process that sets the stage for neurogenesis during corticogenesis. Although there is limited information on the involvement of ATP-dependent chromatin remodelers in this key transition process, there is compiling evidence indicating the importance of chromatin remodelers, especially the BAF complex, in directing proliferation, maintenance, and differentiation of primary NPCs, including aRGs. Hence, as parent cells and source of NPCs, NE cell proliferation and differentiation into aRGs may be regulated by such ATP-dependent chromatin factors during embryonic cortical neurogenesis.

As previously discussed, differential developmental demands during corticogenesis allow assembly of distinct BAF complexes: npBAF complex for progenitor proliferation and nBAF complex for neural progenitor differentiation (Lessard et al., 2007; Staahl et al., 2013; Bachmann et al., 2016). This suggests that, disruption of say key components of npBAF complex can interfere with its function and culminate in aberrant proliferation of NPCs. Indeed, heterozygous loss the SRG3, which is a subunit of the npBAF complex, in mouse was observed to cause abnormal brain development that was attributed to abnormal proliferation of NEs in the germinal zone of the telencephalon (Kim et al., 2001).

Lack of BRG1 in NPCs impaired their proliferation and self-renewal abilities leading to disturbance of neurosphere formation. To that end, brain size was reduced *in vivo* following Nestin-Cre mediated loss of BRG1 in apical NPCs of E10.5 mouse cortex. This phenotype was attributed to reduced proliferation of neural progenitors and diminished pool of neural precursors in such mutant brains (Matsumoto et al., 2006; Lessard et al., 2007). Furthermore, the phenotypic outcomes of knockdown of other BAF complex subunits like BAF45a/b, BAF53a, and BAF55a in NPCs indicate the importance of other BAF complex subunits in proliferation and expansion of progenitors during cortical neurogenesis (Lessard et al., 2007; Staahl et al., 2013).

Indeed, key signaling cascades such as sonic hedgehog, notch signaling, and Wnt-β catenin pathways that are known to regulate proliferation of progenitor cells during neural development have been shown to interact with the BAF complex (Zhan et al., 2011; Vasileiou et al., 2015). The notch signaling pathway, for instance, was observed to be activated by npBAF complex to cause proliferation of neural progenitors during neural patterning, whereas the sonic hedgehog pathway suppressed such proliferative activity under the influence of the BAF complex (Zhan et al., 2011). Also, promotion of telencephalic neural progenitor proliferation by Wnt-β catenin pathways seem to be modulated by BRG1-containing BAF complex (Vasileiou et al., 2015). This implies that manipulating such pivotal signaling pathways in the presence of npBAF complex functionality or vice versa, may provide corrective strategies that can rescue related aberrant cortical phenotypes.

The CHD complex and its close associate, the NuRD complex, appear to be essential in cortical development given the strong and specific expression of some of their subunits in the brain (Thompson et al., 2003; Miccio et al., 2010; Potts et al., 2011). As such, the subunit CHD4 in the NuRD complex (Figure 3C) has been reported to be mostly expressed in neural progenitors during early cortical neurogenesis as opposed to its other family member CHD3, which rather provides ATPase function of the NuRD complex at differentiation stages (Nitarska et al., 2016). For this reason, deletion of CHD4 in apical NPCs, under the control of the Nestin promoter, highlighted its importance in the production and maintenance of apical neural progenitors, including Pax6+/Sox2+ aRGs in the VZ of the developing mouse cortex. In effect, loss of CHD4 in NPCs in the mutant mice (CHD4^fl/fl^/Nestin-CRE) caused reduced proliferative capacity of the CHD4-deficient NPCs at late cortical neurogenesis stages. The decreased proliferation of such NPCs was linked to (i) their precocious exit from the cell cycle, (ii) failure to differentiate, and (iii) eventual cell death (Nitarska et al., 2016). This mainly formed the basis of the reduced cortical thickness observed in the CHD4 mutant mice.

It is therefore not surprising that proliferation of NPCs in the developing brain was also massively decreased when the NuRD complex function was indirectly ablated via disruption of its HDAC domains. Knockout of HDAC2 or chemical inhibition of HDAC in neural progenitors using TSA (Trichostatin A) resulted in blockage of proliferation (Liu et al., 2012; Hagelkruys et al., 2014), but not survival and migration of treated cells (Liu et al., 2012). This consolidates the significance of NuRD complex and/or its associated HDAC1/2 protein functions in finely regulating neural progenitor cell proliferation for proper late-stage differentiative schemes during cortical neurogenesis.

Nevertheless, the ATP-dependent chromatin remodeling factors CHD7 and CHD8 which can function independent of the NuRD complex, have also been implicated in controlling proliferation and maintenance of NEs or NPCs (Hurd et al., 2007; Gompers et al., 2017).

The overall forebrain size is reduced in mice heterozygous for CHD7 (Layman et al., 2011). The telencephalic neuroepithelium appeared dramatically hypoplastic when the developing (E10.5) brain of mice with homozygous loss of CHD7 were examined (Hurd et al., 2007). This suggests that CHD7 may play important role in cortical neurogenesis by exerting its effect early in brain development. Furthermore, several evidences indicating the role of CHD7 in regulating proliferation of olfactory neural stem/progenitor cells (Bosman et al., 2005; Hurd et al., 2007; Layman et al., 2009), neural progenitor maintenance or differentiation in the brain (Bergman et al., 2011; Feng et al., 2017a,b), and adult neurogenesis (Feng et al., 2013), corroborate the plausible role of CHD7 in controlling proliferation of NPCs during corticogenesis.

On the other hand, whereas loss (knockdown) of CHD8 resulted in premature reduction of neural progenitor pool (Durak et al., 2016), deletion of CHD8 in the mouse germline via heterozygous frameshift CHD8 mutation (Chd8^+/del5^) caused increase in NPC proliferation in the developing mouse cortex (Gompers et al., 2017). Mechanistically, reduced expression (haploinsufficiency) of CHD8 in the brain led to aberrant activation of RE-1 silencing transcription factor (REST) which resulted in transcription repression of neuronal genes (Katayama et al., 2016). Chromatin remodeling activity of CHD8 can also regulate the expression of cell cycle genes, the polycomb repressor complex 2 (PRC2), RNA processing factors and inducers of the Wnt/β-catenin signaling pathway (Durak et al., 2016; Gompers et al., 2017). In effect, the delicate balance between the rate of cortical neural progenitor proliferation and differentiation was distorted as a result of CHD8 dysregulation during early corticogenesis; such defect was counterbalanced with β-catenin overexpression in the embryonic (E13–E16) mouse brain and in cultured N2a cells (Durak et al., 2016).

Given that both CHD7 and CHD8 are direct interaction partners and may also be indirectly connected via putative linker proteins (Batsukh et al., 2010), it will be interesting to investigate how their chromatin remodeling activities are coordinated *in vivo* and also the biological consequence of their interactive relationship during cortical development. Such a study promises to elucidate the apparent opposing effect of loss of CHD8 on NPC proliferation as observed in the study conducted by Durak et al. (2016) and Gompers et al. (2017).

MRG15, a stable subunit of the P400/Tip60 chromatin remodeling complex (Figure 3B) and component of the HAT (histone acetyltransferase) and HDAC complexes (Pardo et al., 2002), has been reported to be important in regulating NPC proliferation, maintenance and cell fate determination (Chen et al., 2009, 2011). This may partly be due to its role in regulating transcription, DNA repair, and apoptosis (Squatrito et al., 2006). Lack of MRG15 in the neuroepithelium of E10.5 embryonic brain (MRG15 null mice) rendered it thinner compared to wildtype neuroepithelium. Also, neurosphere formation by cultured NPCs isolated from MRG15-deleted embryonic brain was impaired. BrdU incorporation assay indicated that MRG15 mutagenesis decreased proliferative capacity of MRG15-deficient neural progenitors without affecting their rate of apoptosis *in vitro* (Chen et al., 2009).

Following the above study, the same research group consolidated their claim by showing that MRG15 regulates NPC proliferation by controlling the expression level of cyclin-dependent kinase (Cdk) inhibitor p21 (Chen et al., 2011). Specifically, they noticed that the expression of p21 was up-regulated in NPCs with truncated MRG15 function. For that reason, shRNA-expressing lentiviral plasmid-mediated knockdown of p21 in MRG15 null NPCs was sufficient to rescue their reduced proliferative capacity. As part of the underlying mechanisms, it was also found that activated p53 accumulated in MRG15-deficient NPCs, plausibly underpinning the elevated p21 expression, and making it logical that knockdown of p53 also resulted in restoration of cell proliferation in MRG15 mutant NPCs (Chen et al., 2011).

A cardinal component of the TIP60–p400 complex (Figure 3D) and cofactor of HAT, TRRAP (transformation/transcription domain-associated protein), is known to play specific roles in regulating programs involved in cell-cycle progression of cortical progenitors during neurogenesis. Nestin-Cre-mediated loss of TRRAP in the developing cortex disrupted transcription of E2F cell-cycle target genes through impairment of HAT recruitment and suppression of related transcriptional machinery. This caused cortical NPCs to stay longer in the cell cycle with reduced proliferative capacity that resulted in their untimely differentiation in a cell-autonomous manner (Tapias et al., 2014).

### Regulation of basal cortical progenitor generation and differentiation

Deterministic developmental programs provided by extrinsic and intrinsic factors drive the decision of APs to either proliferate to increase their pool or differentiate into BPs or neurons (Guillemot, 2007a; Kriegstein and Alvarez-Buylla, 2009; Taverna et al., 2014; Tuoc et al., 2014). The role of transcription factors in regulating the generation of BPs during brain development has been extensively investigated. However, relatively little is known about specific epigenetic factors like chromatin remodelers in controlling neural BP generation and differentiation, although such factors are known to regulate chromatin fluidity to alter gene expression patterns. The outcomes of a few studies (see below) in that regard have provided strong evidence indicating important roles played by ATP-dependent chromatin remodeling factors in specifically regulating the genesis of basal neurogenic progenitors and their eventual differentiation during cortical development.

In one such key studies, when the ATP-binding motif of SNF2L (Figure 3C) was conditionally inactivated in mouse brain, it was observed that the head of the resultant mutant (Ex6DEL) was abnormally large. The expanded brain size phenotype was ascribed to excessive amount of cells produced in the Ex6DEL mutant brain. Distinctively, it was found that an unfettered proliferation rate resulted in aberrant increase in Tbr2+ bIP cells in the E15.5 Ex6DEL cortex without any abnormal alteration in the number of Pax6+ apical progenitors. Although neurogenesis in the SNF2L mutant neocortex was temporally disarrayed, the mutant cortex was thick and hypercellular (Yip et al., 2012).

Mechanistically, Yip et al. (2012) found that SNF2L binds to and regulates Foxg1, a transcription factor that regulates NPC self-renewal, basal progenitor expansion and temporal progress of neurogenesis (Siegenthaler and Miller, 2005; Shen et al., 2006; Siegenthaler et al., 2008; Fasano et al., 2009). This implies that dysfunction of Snf2l or related multimeric protein complexes like CERF (Figure 3; Banting et al., 2005) and NURF (Barak et al., 2003) may lead to deregulation of Foxg1 targeting, which further leads to distortion of progenitor cell cycle kinetics, proliferative decisions, and alteration in the timing of neurogenesis in the developing cortex. In an experimental phenotype rescue paradigm, Ex6DEL mutants were crossed with Foxg1 heterozygous mice to generate Ex6DEL:Foxg1^+/−^ mutants with reduced Foxg1 expression in the presence of dysfunctional SNF2L. When E15.5 cortex was examined, it was found that the abnormal Tbr2+ progenitor proliferation phenotype was rescued as result of reduced Foxg1 dosage (Yip et al., 2012). Hence reinforcing the conclusion that SNF2L functions to maintain an appropriate Foxg1 expression level needed for proper basal progenitor generation during cortical neurogenesis.

The BAF complex subunit BAF170 has been reported as an intrinsic factor in regulating the number of basal progenitors in of the neocortex (Tuoc et al., 2013a,b). It was found that mouse cortex-restricted loss of BAF170 promotes Tbr2+ bIP-mediated generation of neurons. Overexpression of BAF170, however, resulted in diminished genesis of Tbr2+ bIP cells, hence promoted direct neurogenesis with associated reductive effect on cortical size due to reduced neuron number. A strong mechanistic detail underlining this phenotype includes the regulation of euchromatin structure due to dynamic competition between the incorporation of BAF170 or its counterpart, BAF155, in the BAF complex. The consequence of this competition dictates the binding effectiveness of Pax6/REST-corepressor regulatory complex to Pax6 gene targets that control the production of bIP cells and late neocortical progenitors. In other words, the genetic interaction between the chromatin remodeling protein BAF170 and Pax6 is critical in determining mouse cortical size via regulation of basal progenitor generation during development (Tuoc et al., 2013a).

Interestingly, deletion of the MBD3/NuRD (methyl binding domain 3/nucleosome remodeling and deacetylation) co-repressor complex also resulted in a reduction in Tbr2+ basal progenitors with attendant phenotype (cortical thickness reduction) quite reminiscent of BAF170 over-expression phenotype during embryonic corticogenesis. The MBD3/NuRD complex was however reported to be dispensable in the requirement of lineage commitment of Pax6+ apical progenitors such as aRGs (Knock et al., 2015).

In a recent study, Moon et al. (2017) showed that Suppressor of Mek null (Smek) interacts with MBD3 to form a critical epigenetic regulatory complex in determining the fate of neural precursor cells during cortical neurogenesis. Double knockout of Smek1 and 2 in mice perturbed cortical neurogenesis such that there was reduced generation Tuj1+/Tbr1+/MAP2+ neurons, whereas the number of Pax6+/Nestin+ progenitors was significantly increased in the early embryonic cortex (Moon et al., 2017). This implies that the increase in Pax6+/Nestin+ progenitors did not translate into increased neuronal output, probably because of inhibition of the production of bIPs that are known to amplify neuronal output from Pax6 or Nestin positive aRGs in the developing cortex. Moreover, even in its predominance, direct neurogenesis may be inadequate for generating enough neurons in the absence of bIP-mediated indirect neurogenesis. Mechanistically, it was reported that Smek facilitates polyubiquitylation and subsequent degradation of MBD3, thereby hampering the formation and recruitment of the MBD3/NuRD co-repressor complex to gene loci whose products are important drivers of neurogenesis (Moon et al., 2017). That implies that Smek functionality promotes acetyl histone H3 activity that in turn augments neuronal differentiation during cortical neurogenesis. As expected, overexpression of MBD3 noticeably stalls differentiation of neurogenic progenitor cells; hence neurogenesis defects consequent to Smek1/2 knockout in mice were significantly rescued by depletion of MBD3 proteins (Moon et al., 2017).

The role of CHD4, and by extension the NuRD complex, is not limited to regulating apical NPC proliferation and maintenance. It was observed that Tbr2+ basal progenitors were significantly reduced at E13.5 and E16.5 in CHD4 mutant mouse brains (CHD4^fl/fl^/Nestin-Cre), with striking reductive effects seen at later developmental stages (Nitarska et al., 2016). It is therefore not far-fetched to reason that such heavy loss of basal neural precursor cells may massively underscore the thin cortical phenotype impacted by CHD4 deletion. This was especially so because the upper cortical laminae, which are predominantly formed by SVZ basal progenitor-derived neurons (Satb2+/Cux1+), were conspicuously reduced as compared to an unchanged number of lower laminae neurons (Tbr1+/Ctip2+) in the cortex devoid of CHD4 expression (Nitarska et al., 2016).

According to Egan et al. (2013), loss of the chromatin remodeler CHD5 in the brain revealed its importance in regulating differentiation of cortical progenitors. Notably, they observed that CHD5 expression was activated in later-stage cortical progenitors and maintained in fully differentiated neurons. This makes it logical that knockdown of CHD5 in progenitors in the V/SVZ impaired neuronal differentiation and led to accumulation of undifferentiated neuronal precursors in the developing neocortex. CHD5 was identified to bind and activate considerable number of genes, including those that orchestrate neuronal differentiation. The study further revealed that neuronal differentiation is likely controlled by the direct interaction of CHD5 with H3K27me3 marks and other Polycomb targets via its chromodomain (Egan et al., 2013).

The importance of TRAPP in regulating cortical neurogenesis appears to include its ability to synchronize timing of cell cycle length of apical progenitors in the VZ of the developing cortex and their differentiation into BPs and neurons (Tapias et al., 2014). Deletion of TRRAP from apical NPCs in the early developing mouse cortex biased their fate toward neuronal and Tbr2+ basal progenitor identity. Interestingly, the unscheduled differentiation of TRRAP-deficient aRG cells to neurons and BPs was rescued by simultaneous gain-of-function of cyclin B1 and A2 (Tapias et al., 2014).

Overall, it is becoming clear that ATP-dependent chromatin remodeling factors and complexes establish regulatory axes together with other proteins to determine the production of basal progenitors through the regulation of their self-renewal and differentiative tendencies during cortical neurogenesis. Perhaps, the differences in the number of basal progenitors and their proliferative capacity in the mouse cortex compared to the primate cortex may be a clue to the existence of plausible differential evolutionary mechanisms or conditions giving rise to the inter-species variation thereof. For instance, it can be argued that the murine ATP-dependent chromatin remodeling factors may be functionally insufficient in causing transcriptional activation of bRG—expression genes like TNC, PTPRZ1, FAM107A, HOPX, and LIFR, which are found only in primate cortices (Fietz et al., 2010; Hansen et al., 2010; Lui et al., 2014; Florio et al., 2015; Pollen et al., 2015; Thomsen et al., 2016). There could also be among other reasons, evolutionary differences in the inheritability of the chromatin remodeling machinery that affords the pattern of BP generation from APs cross species.

One thing that remains unclear, however, is whether such epigenetic chromatin remodelers exclusively sculpt the epigenetic landscape in APs to influence their fate or that their activities linger and/or get modified in their derivatives (BPs) to exert later effects. If the latter is the case, at least as partly implicated in the recent work of Albert et al. (2017), then it would be interesting to investigate the effect of specific loss of such chromatin remodeling factors in specific basal progenitor cells during cortical development. That way, a more comprehensive understanding of how ATP-dependent chromatin remodeling factors epigenetically regulate the generation of various types of basal progenitors in the cortex. For now, the available evidence in that regard remain incomplete, as majority of previous studies only report generalized effects of ATP-dependent chromatin regulators on the generation of all basal progenitors in the telencephalon without specific mention of any subclass.

### Genesis of cortical neuron subtypes

During cortical development, a great number of neurons are generated from different progenitor populations. The vast number of neurons generated during cortical neurogenesis obtain various subtype identities, making it possible to generate (by definition) the six cellularly distinct laminae that typify the mature cortical plate (Molyneaux et al., 2007; Guy and Staiger, 2017).

For instance, the millions of projection neurons (PNs) that are born from progenitors in the germinative zones of the developing cortex are sorted out (molecularly, morphologically, and functionally) through differential activation and deactivations of batteries of developmental cell programs, including transcriptional and epigenetic mechanisms. These afford acquisition of specific identities to yield the typical PNs diversification in the neocortex (Guillemot, 2007b; Yoo and Crabtree, 2009; Hirabayashi and Gotoh, 2010; Narayanan and Tuoc, 2014; Yao et al., 2016; Albert et al., 2017; Sokpor et al., 2017). Thus, this subtype specification underlines the establishment of populations of PNs that specifically projects to subcerebral centers while others make ipsilateral or contralateral hemispheric intracortical connections (Custo Greig et al., 2013; Harb et al., 2016).

The BAF complex has been identified as one of the key molecular factors that regulate neuronal subtype specification during cortical neurogenesis. The BRM ATPase-containing BAF complex has been demonstrated *in vivo* to regulate the formation of upper layer neuronal population during cortical development (Tuoc et al., 2013a,b). Therefore, genes that are expressed by such upper layer neurons (Cux1 and Tle1) were identified to be regulated by the BAF complex in a time-dependent manner via recruitment of Pax6 to bind to such gene targets. As part of the mechanism(s) allowing binding of Pax6 to gene targets to specify upper layer neuronal identity (Tuoc et al., 2009; Georgala et al., 2011), it was reported that the BAF complex subunits BAF155 and BAF170 play important role(s) in the recruitment process (Tuoc et al., 2013a).

Ctip1 and its paralog Ctip2 (BAF100b/Bcl11b) have also been identified to play pivotal roles in neuronal subtype specification during corticogenesis. Whereas Ctip1 is distinctly expressed by post-mitotic cortical neurons that make callosal and corticothalamic connections, Ctip2 is strongly expressed by subcerebral cortical neurons that make corticofugal connections with brainstem nuclei and the spinal cord. In other words, Ctip1 finely regulates cortical neurogenesis through modulation of pathways that lead to deep layer neuron generation, whereas Ctip2 expression or presence in the BAF complex orchestrates the establishment of neurons that make, for example, corticospinal projections (Arlotta et al., 2005; Woodworth et al., 2016).

These factors may however act together with others in determining cortical neuron subtype, as traditionally the case in most developmental pathways. At least it has been shown that the transcription factor Fezf2 critically controls specification of subcerebral PNs during cortical development through regulating the expression of Ctip2. However, both factors synergize functionally to repress the expression of genes like Sox5, Satb2, and Trb1 that lead to the specification of other neuronal subtypes (Arlotta et al., 2005; Chen et al., 2005, 2008; Molyneaux et al., 2005; Bedogni et al., 2010; Cánovas et al., 2015).

The converse also seems to be true. That is, in order to generate different neuronal subclasses during corticogenesis, at least in some instances, Ctip2 and its cofactors have to be co-repressed to allow the developmental of cortical layers as follows: (i) suppression by Tbr1 is needed for the formation of cortical layer 6 (Bedogni et al., 2010; Han et al., 2011), (ii) Sox5-mediated suppression promotes generation of neurons that make layer 5/6 (Kwan et al., 2008; Lai et al., 2008; Shim et al., 2012), and (iii) repression by Satb2 affords the formation of PN subtypes that will form upper cortical layers (Alcamo et al., 2008; Britanova et al., 2008).

Notably, there appears to be no obvious compensatory factor or mechanism that can substitute for the neuronal subclass specification function of Ctip1, and likely Ctip2, or probably the entire chromatin remodeling BAF complex. In line with that, it was observed that there was preponderance of subcerebral neuron generation in sensory areas of the developing cortex, as against specification of deep layer neurons, in the absence of Ctip1 expression, whereas overexpression of Ctip1 suppressed production of subcerebral neurons (Woodworth et al., 2016).

During very early stages of cortical development, the LIM homeodomain transcription factor LHX2 functions as a cortical selector gene to fundamentally specify the cerebral cortex (Mangale et al., 2008; Chou et al., 2009). However, in late embryonic corticogenesis, LHX2 acquires additional function in neuronal subtype identity establishment through the augmentation of the NuRD complex functionality (Muralidharan et al., 2017). The NuRD complex, as a proximal regulator of chromatin dynamics, is reported to interact with LHX2 via its component subunits LSD1, HDAC2, and RBBP4, in order to edit the epigenetic patterns at distal regulatory elements of its target loci: Fezf2 and Sox11, which are known determinants of subcerebral (deep layer) cortical PNs specification (Muralidharan et al., 2017). In the absence or excessive increase of LHX2-NuRD complex interaction, there is an abnormal increase or decrease, respectively, in the population of cortical layer 5 (Fezf2+/Ctip2+) neurons (Muralidharan et al., 2017). This suggests that the NuRD complex is able to alter epigenetic signatures of corticofugal neuron-specifying genes through targeting of Lhx2 to cause appropriate neuronal identity specification in the developing cortex.

To reinforce the essentiality of the NuRD complex in orchestrating specification of neuronal subtype identity, another research group (Knock et al., 2015) reported that deletion of the MBD3 component of the NuRD complex can interfere with proper specification of neocortical PN subtypes. In relation to Satb2+ upper layer neurons, normal proportions of Tbr1+ and Ctip2+ deep layer neurons were seen in MBD3-null cortices at E14.5, but from E16.5 onwards, these neuronal populations were out of proportion such that aberrantly more Tbr1 and Ctip2 expressing neurons compared with Satb2 expressing neurons were seen in the MBD3 cKO cortex (Knock et al., 2015). Cortical mislamination was hence evident in MBD3-deficient cortex since the classical cytoarchitectural layering of neuronal subtypes, as seen in the wildtype cortex, was demonstrably in disarray.

Strikingly, it was observed that cortical neural progenitors that have lost their MBD3/NuRD activity ambiguously express both deep- and upper-layer neuronal markers and hence reflective of some confusion in neuronal lineage selectivity programming during cortical neurogenesis (Knock et al., 2015). This is in consonance with earlier studies suggesting MBD3/NuRD complex as a decisive regulatory factor in the specification of Satb2+ upper layer neurons through the suppression of Ctip2 in Satb2 expressing neurons in the developing cortex (Britanova et al., 2008; Gyorgy et al., 2008). Therefore, the lack of MBD3/NuRD complex activity in mutant mice likely displayed an abnormal temporal extension of deep layer neuron differentiation at the expense of upper layer neurons generation (Knock et al., 2015).

### Migration of cortical neurons

After principal neurons are generated from progenitors in both the VZ and SVZ, they migrate (move) out mainly radially from their birthplaces to their home layers in the cortical plate. Together with default dispositions such as specific time and place of birth, and type of parent progenitor involved, these newly born (immature) cortical neurons are able to collect many regulatory molecular cues in the microenvironment along their migratory trajectory (Evsyukova et al., 2013).

Amongst these regulatory factors, epigenetic regulators, including chromatin remodeling factors are emerging as prominent determinants in ensuring proper placement of neurons after they are born remote to their final position. Until now, one well-documented piece of evidence proving the plausible importance of ATP-dependent remodeling factors in neuronal migration during cortical neurogenesis is the one posited by Wiegreffe et al. (2015). In their study, they showed that Ctip1 is important in regulating how cortical neurons migrate radially during cortical neurogenesis.

It was previously reported that cells in the IZ of the developing cortex strongly express Ctip1 (Leid et al., 2004). Wiegreffe et al. (2015) then advanced the biological significance of the said expression pattern by deleting Ctip1 function via *in utero* electroporation of Cre-GFP plasmid into Ctip1^fl/fl^ E14.5 mouse cortex. This resulted in the accumulation of Ctip1-deficient multipolar neurons in the IZ as compared to the corresponding control. Given that during radial migration multipolar neurons characteristically switch morphology to bipolar neurons so as to migrate properly to their final destination in the CP, the observed stagnation of multipolar neurons in the IZ indicated disruption of the aforementioned critical morphological transition and hence the perturbation of neuronal migration (Wiegreffe et al., 2015). They concluded their investigation by mechanistically associating regulation of the polarity and orientation of radially migrating cortical neurons to Ctip1 and its downstream cofactor Sema3c, to permit normal radial migration known to be key for normal cortical lamination.

Cysteine nitrosylation (S-nitrosylation) of the NuRD complex subunit HDAC2 is known to control its association with chromatin (Nott et al., 2008). During cortical development, S-nitrosylation of HDAC2 at two cysteine residues (Cys262 and Cys274) in neurons is important for activation of specific gene expression programs that regulate radial migration of cortical neurons (Nott et al., 2013). To this end, cortical cells that were electroporated with a mutant form of HDAC2 (HDAC2^C262/274A^), which cannot be nitrosylated at the said cysteine residues, could not migrate out of the IZ to reach the CP. Interestingly, by means of bead-array analysis of the developing cortex, it was observed that S-nitrosylation of HDAC2 activates the expression of the BRM component of the BAF (mSWI/SNF) complex (Nott et al., 2013). Knockout of BRM (BRM^−/−^) caused disruption of radial migration of Cux1+ neurons in the developing cortex, which was a phenocopy of mouse cortical neurons lacking nitric oxide synthase (nNOS^−/−^): the enzyme responsible for S-nitrosylation. Put together, NO signaling seem to cause HDAC2 nitrosylation which in turn regulates the levels of BRM to control radial migration of neurons in the developing cortex (Nott et al., 2013).

The versatility of the CHD/NuRD complex in cortical neurogenesis is again realized in its ability to orchestrate migration of newly born cortical projection neurons. Despite the general similarity in the expression pattern of CHD3 and CHD5, CHD3 is detected in neurons that have reached their home layer in the CP whereas CHD5 expression is observed in the SVZ of the developing cortex (Nitarska et al., 2016), thus possibly depicting their differential role in influencing neuronal differentiation (Egan et al., 2013) and/or migration during cortical neurogenesis.

Indeed, knockdown of CHD3 or CHD5 with short hairpin RNAs (shRNAs) electroporated into the E13.5 cortex affected radial cortical neuron migration when visualized at E18.5. Particularly, CHD3 knockdown caused delay in radial neuronal migration, with significant cell retention in the lower CP as compared with fewer numbers reaching the upper CP. Similarly, knockdown of CHD5 impaired neuronal migration such that many multipolar neurons abnormally accumulated in the IZ, likely reflecting defective multipolar-bipolar state transition, and their overall failure in reaching the CP. Interestingly or perhaps expectedly, loss of CHD4 using either shRNA or Cre-recombinase in CHD4^fl/fl^ cortex did not perturb neuronal migration (Nitarska et al., 2016). This means that the CHD3 and CHD5 components of the NuRD complex are indispensable for proper neuronal migration during cortical neurogenesis, whereas CHD4 functional requirement appears to be reserved for their previously discussed role in neural progenitor genesis.

### Terminal differentiation and maturation of cortical neurons

Following generation and migration of neurons, various differentiation and morphogenetic programs are turned on to ensure attainment of neuronal identity and maturity to permit correct functional neuronal circuitry in the cortex. The elaboration of dendrites (dendritogenesis) or extension of axons (axonogenesis) from neurites are major neuronal maturation events that ensure synapse formation needed for neuronal information processing.

Specific factors, including epigenetic chromatin regulators, have also been identified to play key roles in neuronal terminal differentiation and maturation during neural development (Whitford et al., 2002; Wu et al., 2007). As previous discussed, normally during neural development, npBAF complex respond to differentiation signals by means of subunit reconstitution to produce nBAF complex. Notably, together with other changes, the subunit BAF53a in npBAF complex is switched to BAF53b in the nBAF complex that is strictly functional in post-mitotic neurons (Olave et al., 2002; Lessard et al., 2007; Bachmann et al., 2016). During development of the telencephalon, expression of BAF53b subunit in post-mitotic neurons has been reported to be essential for dendritic arborization and synaptic plasticity (Wu et al., 2007; Vogel-Ciernia et al., 2013; Vogel-Ciernia and Wood, 2014; Choi et al., 2015). Furthermore, it was found that BAF53b-deficient (BAF53b^−/−^) cultured cortical neurons are unable to undergo activity-dependent dendritic outgrowth. Such BAF53b^−/−^ mutant cortical neurons were however able to elaborate dendrites only in the presence of BAF53b functional restoration, but not its homolog BAF53a (Wu et al., 2007). Interestingly, regulation of dendritogenesis during maturation of cortical neurons is not limited to the function of BAF53b but also other nBAF complex subunits like BRG1, BAF45b, and BAF57 (Lessard et al., 2007; Wu et al., 2007).

Ctip1 also plays a vital role in neuronal maturation during embryonic cortical neurogenesis which allows for the formation of thalamocortical axonal connections in the postnatal cortex. Its expression was identified as a regulator of layer 5 cortical neurons maturation needed for their correct integration into appropriate barrel-related column (Greig et al., 2016).

A component of the nBAF complex, BAF55b, also called CREST (calcium-responsive transactivator) or SS18-like protein 1 (SS18L1), has been shown to play an essential role in neuronal morphogenesis. CREST expression is detectable in the developing mouse cortex from E18.5, with peak expression level at P1 and minimal but constant levels from P10 onwards. Activation of CREST is suggested to be a mechanistic aspect of calcium signaling known to regulate development of dendrites during early cortical development (Aizawa et al., 2004). Targeted abolishment of CREST in mouse cortical neurons disrupted calcium-dependent dendritic growth, as revealed by Golgi staining. Such depolarization-induced dendritic elaboration impairment was rescued by overexpression of full-length CREST protein, indicating its cell autonomous function in regulating growth of dendrites during maturation of cortical neurons (Aizawa et al., 2004).

HDAC2 has also been reported to be critical for dendrite development of cortical neurons. The relatively high expression of HDAC2 and its nitrosylation in post-mitotic neurons, as compared to neural progenitors, has been argued to be of importance in regulating dendritic elaboration during neuronal maturation likely via activation of CREB (cyclic-AMP-responsive-element-binding protein)-dependent gene expression pathways (Nott et al., 2008). When S-nitrosylation of HDAC2 was inhibited in embryonic cortical neuron, it led to decrease in dendritic growth and branching. In particular, neurotrophins like brain-derived neurotrophic factor (BDNF) are reported to mediate nitric oxide (NO) signaling that leads to S-nitrosylation of HDAC2 and which ultimately can regulates neuronal dendritogenesis (Nott et al., 2008).

## Conclusion and future perspectives

Neurogenesis in the cortex is a delicately organized developmental event that requires appropriate synchronization of molecular cues leading to proliferation, differentiation, migration, and the ultimate maturation of neurons. The developmental tendency of multipotent apical NPCs to self-renew or differentiate into more fate-restricted derivatives (basal progenitors and neurons), is critically regulated by external and inherent cellular programs that are mainly stimulated by neurogenic transcription and signaling factors. Epigenetic factors are known to implicitly contribute to such regulatory developmental decisions during cortical neurogenesis. Among such epigenetic programs, chromatin modification constitutes a formidable global mechanism used by NPCs to fundamentally adapt their transcriptional response to varying environmental conditions during corticogenesis. More so, extensive remodeling of chromatin architecture permits the sequential transformation of multipotent apical NPCs through specific intermediate precursor cell species into fully differentiated cortical neurons.

By using strategic regulatory mechanisms of action, ATP-dependent chromatin remodelers are able to modulate gene expression programs and other cofactors involved in specific aspects of neurogenic events leading to derivation of neurons from the simple neuroepithelium. The existence of diverse multi-subunit complexes that function as ATP-dependent chromatin remodeling factors may largely depict their unsubstituted requirement in regulating specific parts of cortical neurogenesis rather than providing compensatory functions in the absence or dysfunction of others. Classically, complete or partial inactivation of specific ATP-dependent chromatin remodelers in the developing brain elicit a range of abnormalities such as (i) compromise in neural specification, (ii) up or downregulation of proliferative capacity of apical and basal progenitors, (iii) precocious or delayed differentiation of apical/basal progenitor cells, and (iv) impaired migration and terminal differentiation of post-mitotic neurons. Such aberrant cortical developmental processes are known to culminate into various brain structure and function perturbations.

The increasing number of neurodevelopmental disorders linked to spontaneous or *de novo* mutations in genes coding for chromatin remodeling proteins gives compelling biological significance of stepping up investigative efforts into knowing how ATP-dependent chromatin remodelers regulate cortical neurogenesis. In that direction, applying state-of-the-art tools that can allow us to target and identify associated cofactors and mechanisms involved will help consolidate our understanding of chromatin regulation during brain development in health or disease (Sokpor et al., 2017). For instance, rather than studying the effects of loss of specific ATP-dependent remodeling factors on general population of NPCs (i.e., APs, BPs), it would be more enlightening to determine such consequences on specific progenitor cell types (NEs, aRGs, aIPs, bIPs, and oRGs) in the developing cortex.

The advent of new culture systems for neural cells and transgenic mouse models with cell type-specific reporters, coupled with recently developed proteomic approaches, can allow us determine the cell type-specific composition of each chromatin remodeling complex. Identification of species-specific genes that encode for chromatin remodelers can also be achieved via application of single cell (sc)RNA-seq technique. Furthermore, the newly developed super-resolution nanoscopy coupled with new-labeling methods will provide an additional insight into how chromatin-remodeling factors control chromatin dynamics during neural development. Finally, the application of a robust epigenome-editing technology can afford accurate targeting of chromatin remodeling factors at relevant gene loci to determine their inter- and intra-species gene expression regulatory patterns in the brain.

Altogether, these strategies can permit precise segregation of the heterogeneous cell populations in the developing cortex and identify their unique chromatin remodeling profiles and epigenetic landscapes that specifically contribute to cortical development and evolution.

## Author contributions

GS, RC-H, JR, JS, and TT all contributed to writing and editing the manuscript.

### Conflict of interest statement

The authors declare that the research was conducted in the absence of any commercial or financial relationships that could be construed as a potential conflict of interest.
